# First Australian estimates of incidence and prevalence of uterine fibroids: a data linkage cohort study 2000–2022

**DOI:** 10.1093/humrep/deae162

**Published:** 2024-07-16

**Authors:** L F Wilson, K M Moss, J Doust, C M Farquhar, G D Mishra

**Affiliations:** School of Public Health, The University of Queensland, Herston, QLD, Australia; School of Public Health, The University of Queensland, Herston, QLD, Australia; School of Public Health, The University of Queensland, Herston, QLD, Australia; Department of Obstetrics and Gynecology, University of Auckland, Auckland, New Zealand; School of Public Health, The University of Queensland, Herston, QLD, Australia

**Keywords:** Australia, cohort study, incidence, prevalence, uterine fibroids

## Abstract

**STUDY QUESTION:**

What is the estimated prevalence and incidence of uterine fibroids diagnosed in Australian women of reproductive age?

**SUMMARY ANSWER:**

An estimated 7.3% of Australian women had a diagnosis of uterine fibroids by the age of 45–49 years, with age-specific incidence highest in women aged 40–44 years (5.0 cases per 1000 person-years).

**WHAT IS KNOWN ALREADY:**

Uterine fibroids are associated with a high symptom burden and may affect overall health and quality of life. Studies in different countries show a wide variation in both the prevalence (4.5–68%) and incidence (2.2–37.5 per 1000 person-years) of uterine fibroids, which may be partly explained by the type of investigation, method of case ascertainment, or the age range of the study population, necessitating the reporting of country-specific estimates.

**STUDY DESIGN, SIZE, DURATION:**

This observational prospective cohort study using self-report survey and linked administrative data (2000–2022) included 8066 women, born between 1973 and 1978, in the Australian Longitudinal Study on Women’s Health.

**PARTICIPANTS/MATERIALS, SETTING, METHODS:**

A combination of self-report survey and linked administrative health data (hospital, emergency department, the Medicare Benefits Schedule, and the Pharmaceutical Benefits Scheme) were used to identify women with a report of a diagnosis of uterine fibroids between 2000 and 2022.

**MAIN RESULTS AND THE ROLE OF CHANCE:**

Of the 8066 Australian women followed for 22 years, an estimated 7.3% of women (95% CI 6.9, 7.6) had a diagnosis of uterine fibroids by the age of 45–49 years. The incidence increased with age and was highest in women aged 40–44 years (5.0 cases per 1000 person-years, 95% CI 4.3, 5.7 cases per 1000 person-years). Women with uterine fibroids were more likely to experience heavy or painful periods. They were also more likely to report low iron levels, endometriosis, and poor self-rated health and to have two or more annual visits to their general practitioner.

**LIMITATIONS, REASONS FOR CAUTION:**

Our estimates are based on self-report of doctor diagnosis or treatment for fibroids and/or data linked to treatment and procedure administrative records. This predominantly captures women with symptomatic fibroids, but has the potential for misclassification of asymptomatic women and an underestimate of overall prevalence and incidence. In addition, questions on fibroids were only asked in surveys when women were 37–42 years of age to 43–48 years of age, so cases at younger ages may have been underestimated (particularly in women with less severe symptoms) as these were only ascertained through data linkage.

**WIDER IMPLICATIONS OF THE FINDINGS:**

These are the first population-based estimates of the prevalence and incidence of uterine fibroids in women of reproductive age in Australia. Establishing these first estimates will help inform health policy and health care provision in the Australian context.

**STUDY FUNDING/COMPETING INTEREST(S):**

The ALSWH is funded by the Australian Government Department of Health and Aged Care. L.FW. was supported by an Australian National Health and Medical Research Council (NHMRC) Centres for Research Excellence grant (APP1153420) and G.D.M. was supported by an NHMRC Leadership Fellowship (APP2009577). The funding bodies played no role in the design, the collection, analysis or interpretation of data, the writing of the manuscript, or the decision to submit the manuscript for publication. There are no competing interests.

**TRIAL REGISTRATION NUMBER:**

N/A.

## Introduction

Uterine fibroids (also known as leiomyomas or myomas) are benign tumours that develop from the smooth muscle tissue of the uterus and represent a common gynaecological condition in women of reproductive age (Stewart *et al.*, 2016). While many women with fibroids may be asymptomatic, fibroids can have a significant impact on health status, social and workforce participation, and overall quality of life (Downes *et al.*, 2010; Soliman *et al.*, 2017; Marsh *et al.*, 2018). Common symptoms of fibroids are heavy menstrual bleeding leading to anaemia, and dysmenorrhea, and women may also experience back pain, urinary issues, and bowel issues depending on the number, type, size, and location of the fibroid/s (Fuldeore and Soliman, 2017; Soliman *et al.*, 2017; Petraglia and Dolmans, 2022). Fibroids can also impact fertility and lead to complications in pregnancy (Klatsky *et al.*, 2008; Pritts *et al.*, 2009; Chen *et al.*, 2021).

The biological mechanisms of fibroids are not completely understood; however, sex hormones (mainly oestrogen and progesterone), genetic factors, growth factors, and disordered wound healing (Wise and Laughlin-Tommaso, 2016) are all thought to play a role. Environmental exposures *in utero* and in early life may also be important contributors (Yang *et al.*, 2016).

The strongest established risk factors for fibroids are age and race. Incidence of fibroids increases with age, reaching a peak in women aged 44–54 years, and subsequently declining after menopause (Templeman *et al.*, 2009; Sommer *et al.*, 2015), while the risk of fibroids in Black American women is two to three times higher than the risk in white women (Marshall *et al.*, 1997; Baird *et al.*, 2003; Templeman *et al.*, 2009). Other factors that have been associated with a higher likelihood of fibroids include younger age at menarche, nulliparity, higher BMI, and a family history of uterine fibroids. Women with older age at menarche or who use oral contraceptives are less likely to have uterine fibroids (Templeman *et al.*, 2009; Wise and Laughlin-Tommaso, 2016; Qin *et al.*, 2021; Venkatesh *et al.*, 2022; Song *et al.*, 2023).

Country-specific study estimates of both prevalence and incidence of fibroids vary widely, with prevalences ranging from 4.5% to 68% and incidence rates (IRs) from 217 to 3745 cases per 100 000 person-years in a systematic review of 60 publications (Stewart *et al.*, 2017). Although this variation is potentially due to differences in the type of investigation, method of case ascertainment and the age range of the study population, even when these are similar across studies (Borgfeldt and Andolf, 2000; Templeman *et al.*, 2009), large differences have been found between countries, potentially due to differences in race/ethnicity and/or environmental exposures, indicating that country-specific estimates are required.

Despite being a common benign pelvic tumour in women, population-based incidence and prevalence estimates in Australia have not yet been established. This is a key gap which has implications for both health policy and health care provision. Using data from the Australian Longitudinal Study on Women’s Health (ALSWH), we aimed to estimate the national prevalence and incidence of a reported diagnosis of uterine fibroids over a 22-year period (2000–2022) in a cohort of women born between 1973 and 1978, and to describe the socioeconomic, symptom, and health characteristics of these women by fibroid status.

## Materials and methods

### Study population and participants

The ALSWH is a national longitudinal study that commenced in 1996 and included three cohorts of women born in 1973–1978, 1946–1951, and 1921–1926 to reflect different life stages. Broadly, ALSWH aims to explore factors related to women’s mental and physical health, as well as their use of health services (Lee *et al.*, 2005; Dobson *et al.*, 2015). Details of eligibility and recruitment methods have been previously described. Briefly, participants for each of the cohorts were randomly sampled from the Medicare database (which contains details of all Australian citizens and permanent residents), with women living in rural and remote areas sampled at twice the rate of women in urban areas to allow statistical comparisons between these groups (Lee *et al.*, 2005; Dobson *et al.*, 2015).

This study used survey and linked administrative data from women in the 1973–1978 cohort. The women in this cohort were aged 18–23 years at study recruitment in 1996 and have been subsequently surveyed approximately every 3 years with the ninth survey completed in 2021 (age 43–48 years). Only women who consented to data linkage were included in the analyses.

### Ascertainment of uterine fibroids

Our outcome was the first report of a diagnosis of uterine fibroids identified through a combination of survey and linked administrative data. Linked administrative data sources were: (i) the Medicare Benefits Schedule (MBS) which includes health care services subsidized under Medicare (Australia’s universal health insurance system); (ii) the Pharmaceutical Benefits Scheme (PBS) which includes prescription medicines subsidized under Medicare; (iii) the admitted patient hospital data collections for each State and Territory (public and private hospitals for New South Wales, Victoria, Queensland, and Western Australia; public hospitals only for Australian Captial Territory, Northern Territory, South Australia, and Tasmania); and (iv) public hospital emergency department (ED) admissions data for all States and Territories (except for WA that also includes private ED admissions).

The Australian Institute of Health and Welfare (AIHW) conducts record linkage and extraction for the MBS and PBS. Medicare Personal Identifier Numbers (PINs) for ALSWH participants were validated by Medicare Australia on enrolment to the Study. The AIHW conducts annual deterministic data linkage of ALSWH cohorts, using the Medicare PINs. Checks are also undertaken periodically to investigate any apparent discrepancies. Therefore, the sensitivity of matching for these datasets is considered extremely high. Hospital admissions and ED data are maintained at the State and Territory level. ALSWH data are linked with hospital and ED data using probabilistic linkage based on name, date of birth, address, and address history.

Women were first asked at Survey 7 (2015, aged 37–42 years) whether they had been diagnosed or treated for uterine polyps/uterine fibroids in the previous 3 years. This question was repeated at Survey 8 (2018, aged 40–45 years) and Survey 9 (2021, aged 43–48 years). Because the survey question asked about uterine polyps in addition to uterine fibroids, women who responded ‘yes’ to the survey questions also had to have an MBS, PBS hospital, or ED record for an item that indicated a diagnosis of uterine fibroids. In addition, if a woman answered ‘yes’ to the survey question and had a hospital/ED record for uterine polyps, but no record of uterine fibroids from any linked data source, she was not considered to have uterine fibroids (n = 44). If a woman noted she had uterine fibroids in the open-ended text or comments section of a survey (without a corresponding response to the survey question or a linked data record), then she was considered to have uterine fibroids (n = 15).

If a woman had a record of uterine fibroids from multiple sources, the earliest date was used as the date of diagnosis. The latest date for follow-up of linked data (MBS and PBS collections) was 31 December 2022 (when the oldest women in the cohort were aged 49 years). Because survey questions on uterine fibroids were only asked at Surveys 7, 8, and 9, we only included women who responded to at least one of these surveys in our analysis. Additional details of the survey questions and linked data item codes used to identify cases of uterine fibroids are included in Supplementary Table S1.

### Definition of covariates

To describe the characteristics of women with a diagnosis of uterine fibroids, the following factors were considered. Unless otherwise indicated, all variables were self-report at survey and time dependent, with a woman’s status able to change from survey to survey.

Sociodemographic factors included: age at survey (continuous variable); area of residence categorized according to level of remoteness (Australian Bureau of Statistics, 2018) (‘Major cities of Australia’, ‘Inner regional Australia’, ‘Outer regional/remote/very remote Australia’); highest education level (‘University degree or higher’, ‘Trade/diploma’, ‘High school or less’); and a composite variable derived from questions on country of birth and language spoken at home (asked only at Survey 1 in 1996 and categorized as ‘English-speaking country of birth and mainly English spoken at home’, ‘European country of birth or mainly European language spoken at home’, ‘Other country of birth or mainly other language spoken at home’).

Information on smoking status (‘Non-smoker’, ‘Former smoker’, ‘Current smoker’) was included. Self-report of height and weight was used to calculate BMI and categorized as (‘<25 kg/m^2^’, ’25 to <30 kg/m^2^’, ‘≥30 kg/m^2^’) (World Health Organisation Consultation on Obesity, 1999).

At each survey, women were asked if they had experienced a range of health symptoms in the past 12 months with response options of ‘Never’, ‘Rarely’, ‘Sometimes’, or ‘Often’. We included the following symptoms as three-category variables (‘Never/Rarely’, ‘Sometimes’, ‘Often’): headaches/migraines, severe tiredness, back pain, urinary incontinence, constipation, other bowel symptoms, heavy periods, and painful periods.

Reproductive factors included: age at menarche (asked only at Survey 2 and categorized as ‘≤11 years’, ‘12 years’, ‘13 years’, >13 years’); number of births (‘No births’, ‘One birth’, ‘Two births’, ‘Three or more births’); and current oral contraceptive use (yes/no).

We considered self-report of diagnosis or treatment for low iron levels (iron deficiency or anaemia) and polycystic ovarian syndrome (PCOS). Because information about PCOS was only asked from Survey 4 onwards, values for Survey 4 were also used as Survey 3 responses on the assumption that a woman would have had this condition prior to the first report at Survey 4. A diagnosis/treatment for endometriosis was ascertained using a combination of self-report at survey (Survey 2 to Survey 9) and linked administrative data (see Supplementary Table S2 for survey questions and linked data item codes). Self-report of physical health status, using the General Health Subscale of the Medical Outcomes Study 36-item Short Form health survey (SF-26) and categorized as ‘Excellent/Very good’, ‘Good’, ‘Fair/Poor’, was also included (Ware JE *et al.*, 1993).

Finally, the number of general practitioner (GP) visits in the 12 months prior to completing each survey (ascertained using linked MBS data [Broad Type of Service #101] and categorized into ‘<2 visits/year’, ‘2 to 3 visits/year’, ‘4 to 5 visits/year’, ‘6 or more visits/year’) and whether a woman had one or more visits to a gynaecologist (excluding visits for obstetrics) in the 12 months prior to completing each survey (ascertained using linked MBS data [Service Provider Speciality #53]—and categorized as ‘No visit to gynaecologist’, ‘One or more visits to gynaecologist’) were included.

### Statistical analysis

First, we calculated the prevalence of women with a diagnosis of uterine fibroids. We used the date of the first report of uterine fibroids as a proxy for woman’s age at diagnosis. If the first report was at a survey, we assigned a date that was 18 months prior to completion of the survey as the actual date of diagnosis may have been any time in the 3 years between surveys. Once a woman had a report of uterine fibroids, she was retained as having uterine fibroids at subsequent ages. Women who died (with and without uterine fibroids) were removed from the numerator and denominator at subsequent ages. We calculated prevalence of uterine fibroids by single year of age (data not shown) and then collapsed these into 5-year age groups. Because women from rural and remote areas were over-sampled at recruitment into the 1973–1978 cohort, prevalence estimates were weighted by area of residence (Lee *et al.*, 2005).

Incidence was calculated by identifying the number of new reports of a uterine fibroids diagnosis at each age and dividing that by the estimated total number of years accrued by women at risk of uterine fibroids at the same age, expressed as fibroid cases per 1000 person-years. Women were no longer at risk if they had a previous report of uterine fibroids or had died. As with prevalence, we calculated incidence by single year of age (data not shown) and then collapsed these into 5-year age groups. Incidence estimates were also weighted by area of residence.

In a sensitivity analysis for the estimation of both prevalence and incidence, we maximized the number of women who may have had a diagnosis of uterine fibroids by including women who responded in the affirmative to the survey question about uterine polyps/uterine fibroids but did not also have a linked data record.

To explore the factors associated with incidence of a uterine fibroids diagnosis, we used the date of the first report of fibroids to ascertain the preceding survey and used data from Survey 3 through Survey 8 for the measurement of covariates. We used Survey 3 (2003) as the study baseline due to the small number of women with a first report of uterine fibroids prior to 2003 (n = 5). These women were excluded from our analysis. We did not use data from Survey 9 as there were no fibroid cases with a date of the first report after Survey 9.

We described the baseline characteristics (Survey 3) of women according to whether they had ever had a report of uterine fibroids during the follow-up period (2003–2022). Percentages for descriptive characteristics were weighted by area of residence. We then used Poisson regression using generalized estimating equations with robust error variance to account for repeated measures to calculate rate ratios (RR) with 95% CIs for the associations between the considered factors and incidence of uterine fibroids. The model was adjusted for age, survey, and weighted for area of residence. A woman was excluded from subsequent survey timepoints if she was no longer at risk of uterine fibroids (i.e. had a prior report of uterine fibroids, gave a self-report of having a hysterectomy at a prior survey or died between surveys).

In the regression analyses, uterine fibroids cases were only included if there was a response to the survey preceding the date of diagnosis (469 cases). To explore the impact of excluding cases due to survey non-response (91 cases), in a sensitivity analysis, we imputed missing survey data using the 2-fold fully conditional specification algorithm method for longitudinal data (50 imputations) which imputes missing values at a given survey, conditional on information at the same survey and immediately adjacent surveys (Welch *et al.*, 2014).

All analyses were done using SAS software, Version 9.4 (TS1M6) of the SAS system for Windows Copyright © 2016 by SAS Institute Inc (Carey), except for the multiple imputation which was done in Stata/SE 14 for Windows (StataCorp, 2015).

### Ethical approval

The ALSWH survey program has ongoing ethical approval from the Human Research Ethics Committees (HRECs) of the Universities of Newcastle and Queensland (approval numbers H076-0795 and 2004000224, respectively, for the 1973–1978, 1946–1951, and 1921–1926 cohorts; and H-2012-0256 and 2012000950, for the 1989–1995 cohort). All participants consented to join the study and are free to withdraw or suspend their participation at any time with no need to provide a reason.

The ALSWH also maintains institutional HREC approvals for external record linkage (approval numbers H-2011-0371 and 2012000132, for the Universities of Newcastle and Queensland respectively). In addition, access to MBS and PBS data collections is approved by the Australian Institute of Health and Welfare HREC. Access to state and territory Hospital Admissions and Emergency Department data collections is approved by an appropriate HREC for each jurisdiction.

## Results

Between 2000 and 2022, 565 women had a report of a diagnosis of uterine fibroids from survey and linked data sources. Of these, 71% were identified through a combination of linked data and survey data, 26% were identified through linked data only (hospital or ED data or a combination of hospital/ED and PBS/MBS data), and the remaining 3% were identified solely through specific mention of uterine fibroids in the survey comments/text fields (Supplementary Fig. S1). The most common procedures for a hospital admission for fibroids were myomectomy, insertion of IUD, endometrial ablation, and hysterectomy.

### Prevalence and incidence

**Figure 1.**
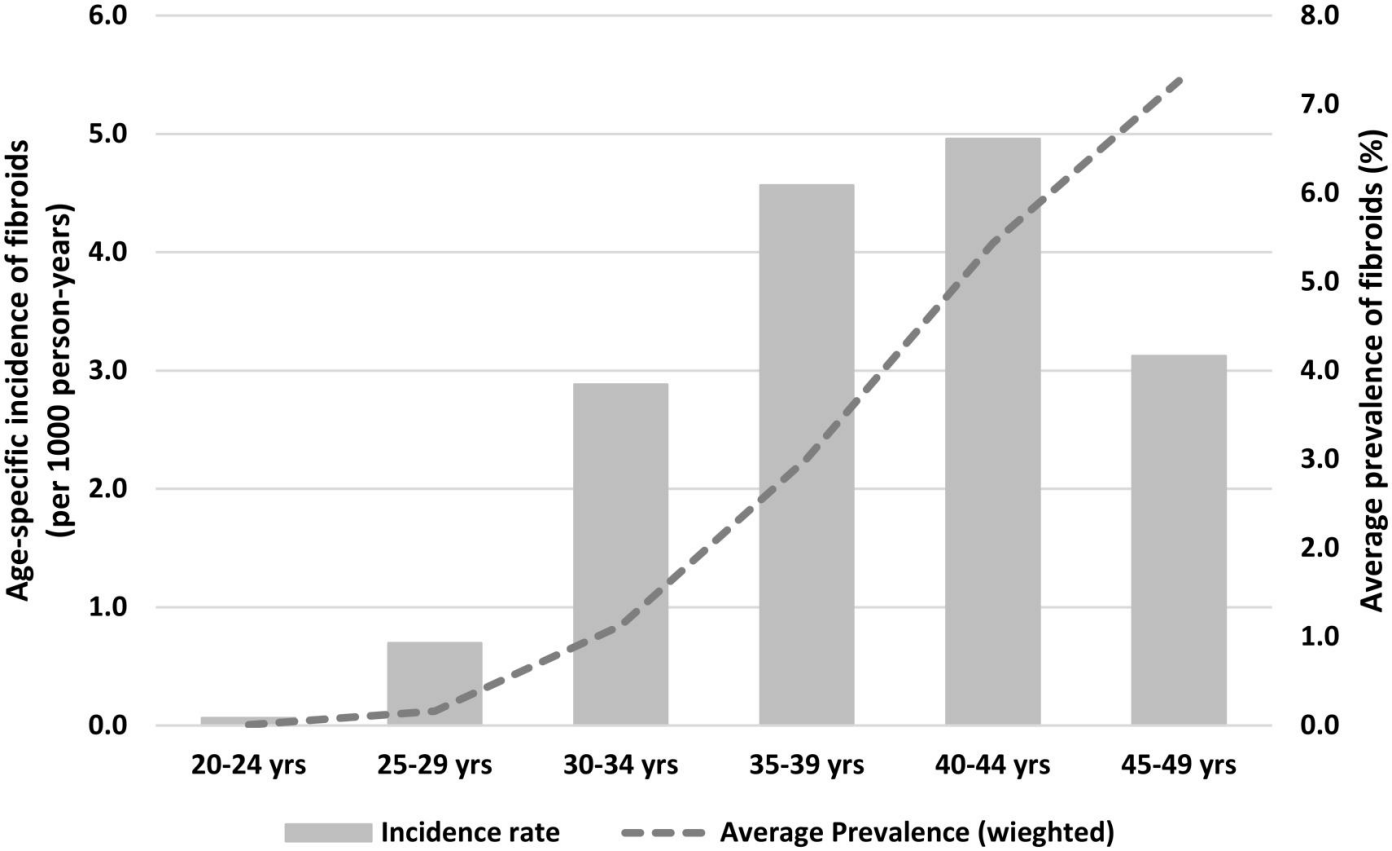
Incidence and prevalence of diagnosed fibroids by 5-year age group, 1973–1978 cohort of the Australian Longitudinal Study on Women’s Health (total n = 8066, fibroids = 565).

The prevalence of diagnosed uterine fibroids was <1% before age 30, increasing to 7.3% (95% CI 6.9, 7.6) by 45–49 years (Fig. 1, dashed line). The incidence of diagnosed uterine fibroids was 0.06 (95% CI 0.02, 0.25) per 1000 person-years at 20–24 years, peaking at 5.0 (95% CI 4.3, 5.7) per 1000 person-years at 40–44 years (Fig. 1, grey bars). Exact numbers for prevalence and incidence of women with a diagnosis of uterine fibroids are included in Supplementary Information (Supplementary Tables S3 and S4). In our sensitivity analysis that included additional fibroid cases by also counting women who had an affirmative response to the survey question on diagnosis or treatment for uterine polyps/uterine fibroids but did not have a linked data record (an extra 182 cases—total 747 cases), the prevalence of diagnosed fibroids was 9.7% (95% CI 9.3, 10.1) by age 45–49 years, and IRs were highest in women aged 40–44 years (7.3, 95% CI 6.5, 8.2 per 1000 person-years; see Supplementary Tables S5 and S6).

### Factors associated with diagnosed uterine fibroids

**Figure 2.**
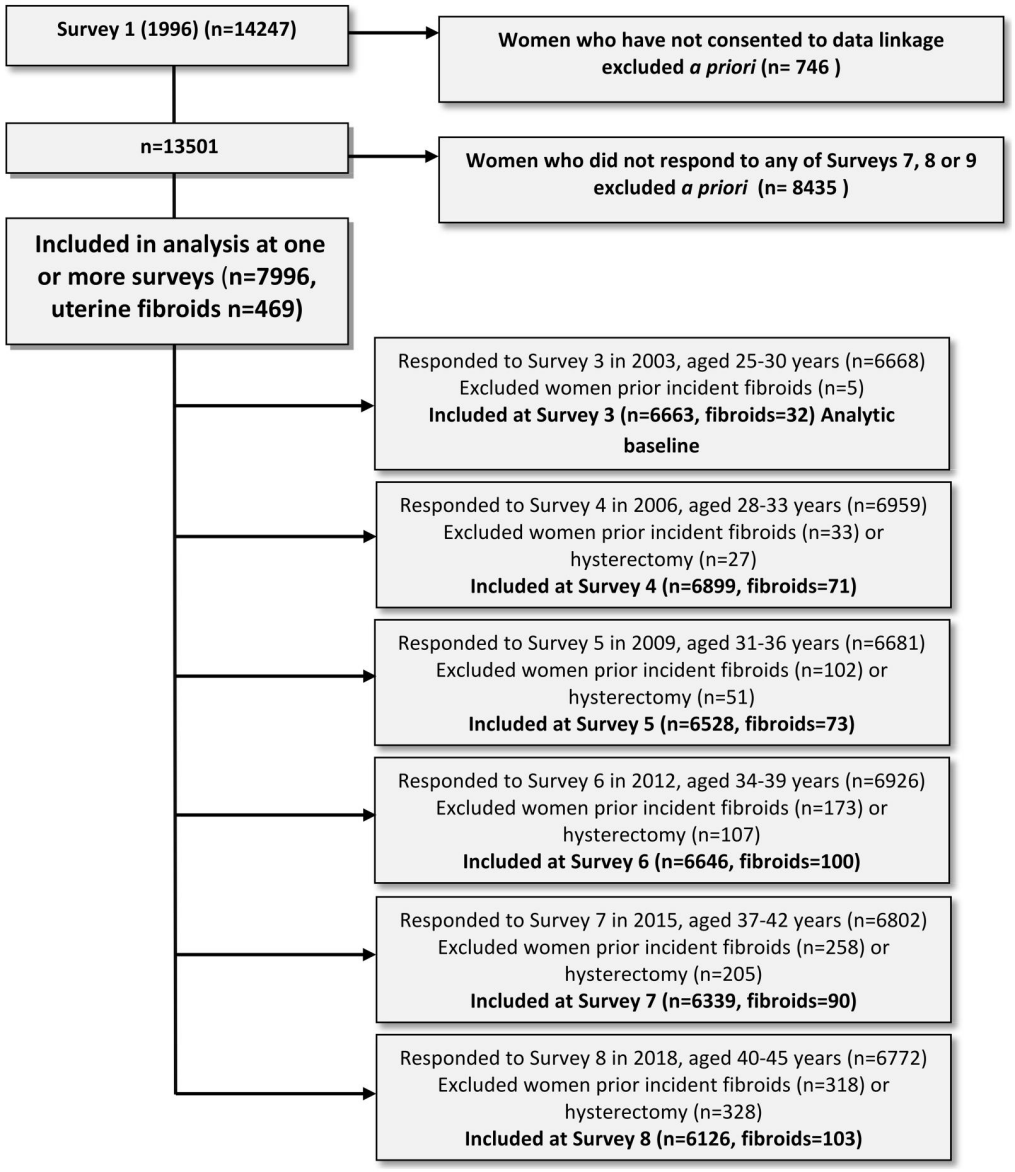
Flow diagram of participants included at each survey in the regression analysis looking at associations between sociodemographic, behaviour, and health factors, and the incidence of uterine fibroids (1973–1978 cohort of the Australian Longitudinal Study on Women’s Health).

The analyses looking at associations between sociodemographic, behaviour, and health factors and diagnosed uterine fibroids included 7996 women. Figure 2 shows the number of women (and incident fibroids cases) included at each survey and the number excluded due to prior incident fibroids or hysterectomy.

Table 1 compares baseline characteristics of women by uterine fibroids status (ever/never having a diagnosis of uterine fibroids) and Table 2 shows the results of the regression analyses.

**Table 1.** Baseline characteristics (Survey 3, 2003 25–30 years) of study population by whether or not women had a record of uterine fibroids during the following up period (Surveys 3–8) of the 1973–1978 cohort of the Australian Longitudinal Study on Women’s Health.

		Record of uterine fibroids (n = 6663)		
		No (n = 6245)	**Yes (n = 418)** ^a^	
		**n** ^b^ **(%)**^c^	**n** ^b^ **(%)**^c^	*P*-value
*Age (years)*	Mean (SD)	27.1 (1.45)	27.2 (1.46)	0.823
*Area of residence*	Major cities	3576 (68.8)	244 (71.0)	0.634
	Inner regional	1619 (18.4)	104 (17.1)	
	Outer regional/rural/remote	1043 (12.8)	70 (11.9)	
*Highest qualification level*	Degree or higher	3026 (53.6)	194 (51.3)	0.602
	Trade/diploma	1564 (23.6)	101 (23.7)	
	High school or less	1642 (22.8)	121 (24.9)	
*County of birth × language spoken at home*	English-speaking country of birth and English-speaking at home	5784 (91.2)	432 (90.4)	0.789
	European country of birth or speaks European language at home	273 (5.5)	22 (5.7)	
	Other country of birth or other language spoken at home	165 (3.3)	14 (3.9)	
*Headaches/migraines*	Never/Rarely	2837 (46.4)	174 (40.7)	0.061
	Sometimes	2326 (36.6)	159 (38.3)	
	Often	1079 (17.0)	85 (21.0)	
*Severe tiredness*	Never/Rarely	3540 (57.1)	217 (53.5)	0.105
	Sometimes	1787 (28.8)	122 (28.3)	
	Often	915 (14.1)	79 (18.1)	
*Back pain*	Never/Rarely	3827 (61.9)	248 (60.4)	0.063
	Sometimes	1687 (26.9)	106 (24.4)	
	Often	728 (11.2)	64 (15.2)	
*Leaking urine*	Never/Rarely	5828 (93.7)	387 (92.7)	0.275
	Sometimes	341 (5.2)	23 (5.3)	
	Often	73 (1.1)	8 (2.0)	
*Constipation*	Never/Rarely	5091 (81.5)	353 (85.4)	0.150
	Sometimes	890 (14.2)	50 (11.7)	
	Often	261 (4.3)	15 (2.9)	
*Other bowel problems*	Never/Rarely	5640 (90.0)	372 (88.3)	0.406
	Sometimes	358 (5.9)	27 (7.6)	
	Often	244 (4.1)	19 (4.1)	
*Heavy periods*	Never/Rarely	5278 (83.9)	302 (72.5)	<0.0001
	Sometimes	636 (10.2)	81 (19.7)	
	Often	383 (5.9)	35 (7.8)	
*Painful periods*	Never/Rarely	4842 (77.7)	274 (63.8)	<0.0001
	Sometimes	844 (13.8)	90 (23.1)	
	Often	556 (8.5)	54 (13.1)	
*Smoking status*	Non-smoker	3704 (60.2)	252 (60.8)	0.860
	Former smoker	1156 (18.1)	80 (18.7)	
	Current smoker	1383 (21.7)	86 (20.5)	
*BMI*	<25 kg/m^2^	3961 (66.5)	222 (53.8)	<0.0001
	25–29.9 kg/m^2^	1324 (20.6)	113 (28.8)	
	≥30 kg/m^2^	847 (12.9)	79 (17.4)	
*Age at menarche*	≤11 years	701 (12.8)	83 (21.0)	<0.0001
	12 years	1504 (27.3)	120 (28.0)	
	13 years	1707 (30.7)	112 (28.8)	
	>13 years	1605 (29.3)	93 (22.2)	
*Current oral contraceptive use*	No	3247 (51.9)	248 (60.7)	0.001
	Yes	2991 (48.1)	169 (39.3)	
*Endometriosis*	No	5877 (94.4)	375 (90.2)	0.001
	Yes	368 (5.6)	43 (9.8)	
*Polycystic ovarian syndrome*	No	5161 (94.9)	337 (89.9)	<0.001
	Yes	266 (5.1)	34 (10.1)	
*Low iron levels*	No	5314 (85.3)	334 (79.7)	0.005
	Yes	921 (14.7)	83 (20.3)	
*Self-rated health*	Excellent/Very good	3628 (59.0)	201 (49.6)	<0.001
	Good	2096 (32.9)	159 (37.2)	
	Fair/poor	519 (8.1)	58 (13.2)	
*Annual GP visits*	Less than 2 visits	1606 (25.5)	80 (18.6)	0.002
	2–3 visits	1830 (29.1)	112 (26.0)	
	4–6 visits	1229 (19.5)	102 (25.0)	
	More than 6 visits	1628 (25.7	124 (30.4)	
*Annual gynaecologist visits*	No visits	5246 (84.2)	345 (83.7)	0.805
	1 or more visits	999 (15.8)	73 (16.3)	

a 51 fibroids cases excluded because participant did not respond to Survey 3.

b Numbers for each characteristic will differ due to missing values.

c Percents weighted by area of residence.

**Table 2.** Associations between socioeconomic factors, symptoms, reproductive factors, health factors, and health service use, and incidence of fibroids (2003–2022) in the 1973–1978 cohort of the Australian Longitudinal Study on Women’s Health (n = 8066).

		**RR (95% CI)** ^a^
*Age (years)*		1.06 (0.99, 1.12)
*Area of residence*	Major cities	Ref.
	Inner regional	1.03 (0.84, 1.27)
	Outer regional/rural/remote	0.95 (0.73, 1.24)
*Highest qualification level*	Degree or higher	Ref.
	Trade/diploma	1.05 (0.84, 1.30)
	High school or less	1.13 (0.88, 1.44)
*Country of birth × language spoken at home*	English-speaking country of birth and English spoken at home	Ref.
	European country of birth or European language spoken at home	1.13 (0.73, 1.74)
	Other country of birth or other language spoken at home	1.11 (0.65, 1.89)
*Headaches/migraines*	Never/Rarely	Ref.
	Sometimes	1.23 (1.01, 1.50)
	Often	1.36 (1.05, 1.76)
*Severe tiredness*	Never/Rarely	Ref.
	Sometimes	1.31 (1.06, 1.60)
	Often	1.47 (1.16, 1.87)
*Back pain*	Never/Rarely	Ref.
	Sometimes	1.19 (0.98, 1.45)
	Often	1.33 (1.02, 1.73)
*Leaking urine*	Never/Rarely	Ref.
	Sometimes	0.96 (0.72, 1.27)
	Often	1.77 (1.21, 2.59)
*Constipation*	Never/Rarely	Ref.
	Sometimes	0.93 (0.72, 1.19)
	Often	1.17 (0.77, 1.79)
*Other bowel problems*	Never/Rarely	Ref.
	Sometimes	1.24 (0.91, 1.70)
	Often	1.97 (1.37, 2.85)
*Heavy periods*	Never/Rarely	Ref.
	Sometimes	2.58 (2.08, 3.21)
	Often	4.07 (3.29, 5.05)
*Painful periods*	Never/Rarely	Ref.
	Sometimes	2.70 (2.18, 3.33)
	Often	3.81 (3.02, 4.83)
*Smoking status*	Non-smoker	Ref.
	Former smoker	1.02 (0.83, 1.26)
	Current smoker	1.13 (0.86, 1.48)
*BMI*	<25 kg/m^2^	Ref.
	25–29.9 kg/m^2^	1.06 (0.85, 1.34)
	≥30 kg/m^2^	1.43 (1.15, 1.78)
*Age at menarche*	≤11 years	1.47 (1.12, 1.95)
	12 years	Ref.
	13 years	0.84 (0.65, 1.08)
	>13 years	0.73 (0.56, 0.96)
*Number of births*	No births	1.36 (1.06, 1.74)
	1 birth	1.35 (1.03, 1.78)
	2 births	Ref.
	3 or more births	0.91 (0.79, 1.21)
*Current oral contraceptive use*	No	Ref.
	Yes	0.98 (0.79, 1.21)
*Endometriosis*	No	Ref.
	Yes	2.03 (1.60, 2.57)
*Polycystic ovarian syndrome*	No	Ref.
	Yes	1.63 (1.22, 2.18)
*Low iron levels*	No	Ref.
	Yes	1.84 (1.50, 2.25)
*Self-rated health*	Excellent/Very good	Ref.
	Good	1.55 (1.27, 1.88)
	Fair/poor	2.25 (1.73, 2.93)
*Annual GP visits*	Less than 2 visits	Ref.
	2–3 visits	1.70 (1.28, 2.26)
	4–6 visits	1.76 (1.31, 2.38)
	More than 6 visits	1.88 (1.42, 2.48)
*Annual gynaecologist visits*	No visits	Ref.
	1 or more visits	1.34 (1.08, 1.68)

a Adjusted for age, survey, and area of residence.

RR, rate ratios.

Women who reported often experiencing a range of symptoms (including headaches/migraines, severe tiredness, back pain, leaking urine, bowel problems, heavy periods, and painful periods) were more likely to have a diagnosis of uterine fibroids compared to women who reported never/rarely experiencing these symptoms (Table 2). The strongest associations were for women who often experienced heavy periods or painful periods (RR 4.07, 95% CI 3.29, 5.05 and RR 3.81, 95% CI 3.02, 4.83, respectively, compared to never/rarely experiencing these symptoms). Women with endometriosis (RR 2.03, 95% CI 1.60, 2.57), PCOS (RR 1.63, 95% CI 1.22, 2.18) or low iron levels (RR 1.84, 95% CI 1.50, 2.25) were also more likely to have diagnosed uterine fibroids. Women who rated their health as fair or poor (compared to those who thought they were in excellent health) were more likely to have diagnosed fibroids (RR 2.25, 95% CI 1.73, 2.93) as were women who had more annual GP visits, or at least annual visits to a gynaecologist. Compared to women who reported menarche at 12 years of age, women with menarche <11 years were more likely to have diagnosed fibroids (RR 1.47, 95% CI 1.12, 1.95), as were women with fewer than two births. None of the included sociodemographic factors were associated with a diagnosis of uterine fibroids. In the sensitivity analysis that used imputed missing survey data, the interpretation of the results remained the same (Supplementary Table S7).

## Discussion

In this longitudinal study of Australian women born between 1973 and 1978, an estimated 7% of women had a diagnosis of uterine fibroids by the age of 45–49 years. IRs were highest in the 40- to 44-year age group at 5.0 cases per 1000 person-years. Women who reported they often experienced heavy or painful periods, or had low iron levels or endometriosis were more likely to have a diagnosis of uterine fibroids. Women with fibroids were also more likely to have poorer self-rated health and to have two or more annual visits to their GP.

There is high variation in the country-specific prevalence of fibroids. A cross-sectional survey of US women reported prevalence of fibroids in white women aged 45–49 years was 9.5% (95% CI 8.5, 10.6) (Fuldeore and Soliman, 2017), while a cross-sectional international internet-based survey of >21 000 women across eight countries, reported considerable inter-country variability in the prevalence of fibroids in women aged 40–49 years, ranging from 9.4% in the UK to 17.8% in Italy (Zimmermann *et al.*, 2012). Prevalence in a Brazilian cross-sectional study of university employees was even higher at 22.7% and 38.6% in women aged 35–44 and 45–54 years, respectively (Boclin Kde and Faerstein, 2013). In all three studies, fibroid cases were ascertained by self-report of a doctor/health professional diagnosis.

Prevalence estimates in our primary analysis (7.3% (95% CI 6.9, 7.6) by age 45–49 years) are lower than all of these studies, although the sensitivity analysis estimates that additionally included diagnoses based on self-report only (9.7% (95% CI 9.3, 10.1) by age 45–49 years) are similar to studies reporting estimates for the USA (Fuldeore and Soliman, 2017) and the UK (Zimmermann *et al.*, 2012). While we used a combination of survey and linked administrative data to ascertain our fibroid cases, our prevalence estimates may be lower because we may have missed cases at younger ages as survey questions about uterine fibroids were first asked at Survey 7 (when women were aged 37–42 years). As the ALSWH sample is predominantly from Australia or other English-speaking countries, differences in the ethnicities of the study populations may also play a role. It is also plausible that diversity in health systems, along with variation in funding, and levels of access to treatments for uterine fibroids may lead to variability in prevalence estimates across countries.

Our IRs were highest in women aged 40–44 years (IR 5.0, 95% CI 4.3, 5.7 per 1000 person-years). Our IR was much lower than those reported in women aged 40–44 years in the Black Women’s Health Study (IR 39.8, 95% CI 36.5, 43.4 per 1000 person-years) (Wise *et al.*, 2005) and the Nurses’ Health Study II (IR 16.0, 95% CI 15.0, 17.0 per 1000 person-years) (Marshall *et al.*, 1997), with fibroid cases ascertained by self-report of a diagnosis that had been confirmed by ultrasound or hysterectomy in both studies. Another US study found similar age-specific IRs for women aged 40–44 years to those of the Nurses’ Health Study II (IR 190.5 per 10 000 person-years ∼19.1 per 1000 person-years) with cases identified through health care records (Yu *et al.*, 2018). Again, our lower IRs in women of the same age may be due to the ethnic differences in the study populations. In contrast, IRs reported in the California Teachers Study were lower than in our study (IR 412.7 per 100 000 women ∼4.13 per 1000 women); however, fibroid cases were ascertained using the more restrictive definition of a principal surgical diagnosis using hospital patient discharge records (Templeman *et al.*, 2009). Our rates were also higher than those of a UK study that used Read codes from general practice data to identify fibroid cases (IR 3.66 per 1000 person-years in women aged 40–44 years), although the reason for this difference is unclear (Martín-Merino *et al.*, 2016).

In our analyses describing the characteristics of women with fibroids, the results of our analyses were consistent with other studies that have shown that menstrual symptoms along with symptoms of severe tiredness, back pain, and bowel issues are more likely to be experienced by women with fibroids (Drayer and Catherino, 2015; Fuldeore and Soliman, 2017; Soliman *et al.*, 2017). The associations with higher BMI (Qin *et al.*, 2021), age at menarche (Wise *et al.*, 2004; Song *et al.*, 2023), fewer births (Pritts *et al.*, 2009), poorer overall health (Downes *et al.*, 2010), low iron levels (Fuldeore and Soliman, 2017), endometriosis (Uimari *et al.*, 2011), PCOS (Fuldeore and Soliman, 2017), and a higher frequency of doctor visits (Laberge *et al.*, 2016) have also been substantiated elsewhere.

### Strengths and limitations

Strengths of our study include the large community-based sample and the availability of a combination of survey and linked administrative data that covered a period of 22 years (with 95% of women in the cohort consenting to data linkage). In addition, the survey questions used to measure the sociodemographic, symptomatic, and reproductive and health variables were repeated at each survey, so the changes in these characteristics over time were accounted for.

A key limitation of our study is that we may have misclassified women whose fibroids were asymptomatic or undiagnosed and may have missed cases at younger ages as survey questions about uterine fibroids were first asked at Survey 7 (when women were aged 37–42 years). Some cases may also have been missed as private hospital admissions data were not available for women living in the Northern Territory, Australian Capital Territory, South Australia, or Tasmania (although these are likely to be small in number as these are the least populous jurisdictions). Because the survey question used to ascertain a diagnosis of uterine fibroids also included uterine polyps, in our primary analysis a record of uterine fibroids identified through survey questions had to be supported by a linked MBS, PBS, or hospital/ED record indicating uterine fibroids. In a sensitivity analysis, when we included all affirmative responses to the survey questions, prevalence estimates were 2% higher than in our primary analysis. While a genetic component has been established for fibroids, neither genetic samples nor questions on race/ethnicity or having a family history of fibroids are available in ALSWH so we could not explore whether these factors were associated with a diagnosis of fibroids in our study population. Finally, the women in our study are more highly educated and more likely to be born in Australia than the general population (Dobson *et al.*, 2015), which may limit the generalizability of the findings to women with lower education levels or diverse cultural backgrounds.

## Conclusions

In a national cohort of Australian women of reproductive age, ∼1 in 14 women had a recorded diagnosis of uterine fibroids. Establishing these first estimates will help inform health policy and health care provision in the Australian context. Repeating these estimates of prevalence and incidence over the next 10 years as the women in our study become post-menopausal will be important as there are limited longitudinal studies that have been able to look at the burden of uterine fibroids in the post-reproductive stages.

## Supplementary Material

deae162_Supplementary_Figure_S1

deae162_Supplementary_Table_S1

deae162_Supplementary_Table_S2

deae162_Supplementary_Table_S3

deae162_Supplementary_Table_S4

deae162_Supplementary_Table_S5

deae162_Supplementary_Table_S6

deae162_Supplementary_Table_S7

## Data Availability

ALSWH survey data are owned by the Australian Government Department of Health and due to the personal nature of the data collected, release by ALSWH is subject to strict contractual and ethical restrictions. Ethical review of ALSWH is by the Human Research Ethics Committees at The University of Queensland and The University of Newcastle. De-identified data are available to collaborating researchers where a formal request to make use of the material has been approved by the ALSWH Data Access Committee. The committee is receptive of requests for datasets required to replicate results. Information on applying for ALSWH data is available from https://alswh.org.au/for-data-users/applying-for-data/. In addition, linked administrative data have been provided by third parties. In order for these linked data to be accessed through ALSWH, every data user must be added to the applicable Data Use Agreements and Human Research Ethics Committee protocols.
