# Supplementary material for: First Australian estimates of incidence and prevalence of uterine fibroids: a data linkage cohort study 2000–2022
Source: Hum Reprod. 2024 Jul 16;39(9):2134–43. doi: 10.1093/humrep/deae162 (PMC11373412; doi:10.1093/humrep/deae162)
Supplement: deae162_Supplementary_Figure_S1 [file deae162_supplementary_figure_s1.pdf]

OXFORD
human reproduction
Human Reproduction, 2024, 1–1
https://doi.org/10.1093/humrep/deae162
Supplementary Data

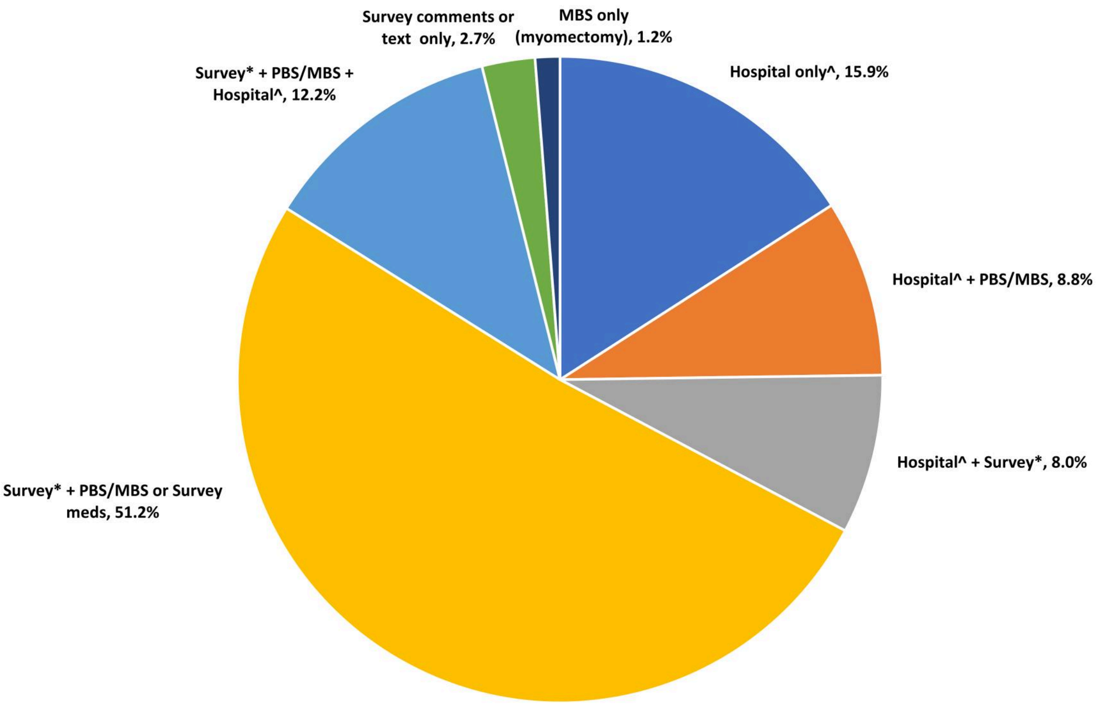

Supplementary Figure S1. Percentage of uterine fibroids cases identified by different combinations of survey and linked data sources in the 1973–1978 cohort of the Australian Longitudinal Study of Women's Health between 2000 and 2022 (n = 565). ^Including Emergency Department data. *Including comments, text fields, and self-reported medications. PBS, Pharmaceutical Benefits Scheme; MBS, Medicare Benefits Schedule.
