# Supplementary material for: First Australian estimates of incidence and prevalence of uterine fibroids: a data linkage cohort study 2000–2022
Source: Hum Reprod. 2024 Jul 16;39(9):2134–43. doi: 10.1093/humrep/deae162 (PMC11373412; doi:10.1093/humrep/deae162)
Supplement: deae162_Supplementary_Table_S1 [file deae162_supplementary_table_s1.pdf]

OXFORD
human reproduction

Human Reproduction, 2024, 1–1
https://doi.org/10.1093/humrep/deae162
Supplementary Data

Supplementary Table S1. Sources of data and linked data codes used to identify women with uterine fibroids in the 1973–1978 cohort of the Australian Longitudinal Study on Women's Health.

Data source	Eligibility criteria and applicable items and codes
ALSWH Surveys	Text response (i.e. specific mention in the free text or comments fields)<br>Text searched:<br>'adenomyo', 'fibroids', 'hysterect', 'leiomyo', 'menorrhagia', 'myoma', 'polycythem', 'uterine'.<br>**OR**<br>Affirmative response to survey question AND one or more of applicable MBS, PBS, Hospital or Emergency Department codes (listed below)<br>Survey question (asked at Survey 7 in 2015, Survey 8 in 2018, and Survey 9 in 2021):<br>'*In the last 3 years, have you been diagnosed with or treated for Uterine polyps/uterine fibroids?*'
Medicare Benefits Schedule (MBS)	Reported once or more:<br>MBS item 35410: UTERINE ARTERY CATHETERISATION with percutaneous administration of occlusive agents, for the treatment of symptomatic uterine fibroids in a patient who has been referred for uterine artery embolization by a specialist gynaecologist, excluding associated radiological services or preparation, and excluding aftercare (from November 2006 to present)<br>MBS item 35649: MYOMECTOMY: one or more myomas, when undertaken by an open abdominal approach (H)
Pharmaceutical Benefits Schedule (PBS)	Only used in combination with ALSWH surveys:<br>Anatomical Therapeutic Chemical (ATC) classification codes:<br>B02AA02 Tranexamic acid<br>G02BA03 Plastic (IUD) with progestogen (Mirena)
Hospital/Emergency Department	Reported once or more OR used in combination with ALSWH Surveys:<br>International Classification of Diseases (ICD) version and diagnosis codes used:<br>ICD-9-CM 218 Uterine leiomyoma<br>ICD-10-AM D25 Leiomyoma of uterus<br>ICD-10-AM 034.1 Maternal care for benign tumour of corpus uteri, uterine fibroids
