# Supplementary material for: First Australian estimates of incidence and prevalence of uterine fibroids: a data linkage cohort study 2000–2022
Source: Hum Reprod. 2024 Jul 16;39(9):2134–43. doi: 10.1093/humrep/deae162 (PMC11373412; doi:10.1093/humrep/deae162)
Supplement: deae162_Supplementary_Table_S2 [file deae162_supplementary_table_s2.pdf]

OXFORD
human reproduction

Human Reproduction, 2024, 1–1
https://doi.org/10.1093/humrep/deae162
Supplementary Data

Supplementary Table S2. Sources of data and linked data codes used to identify women with endometriosis in the 1973–1978 cohort of the Australian Longitudinal Study on Women's Health.

Data source	Eligibility criteria and applicable items and codes
ALSWH Surveys	Survey questions (asked at all surveys from Survey 2 (2000) to Survey 9 (2021)):<br>Survey 2:<br>*'Have you ever been told by a doctor that you have endometriosis?'*<br>Response options: 'In the last 4 years' or 'More than 4 years ago'<br>Survey 3 through to Survey 9:<br>*'In the last 3 years, have you been diagnosed with or treated for endometriosis?'*
Medicare Benefits Schedule (MBS)	Reported once or more:<br>MBS item 35641: Severe endometriosis, laparoscopic resection of, involving 2 of the following procedures:<br>a) resection of the pelvic side wall including dissection of endometriosis or scar tissue from the ureter;<br>b) resection of the Pouch of Douglas;<br>c) resection of an ovarian endometrioma greater than 2 cm in diameter;<br>d) dissection of bowel from uterus from the level of the endocervical junction or above (H)<br>(from November 2000 to present)
Pharmaceutical Benefits Schedule (PBS)	Reported once or more:<br>Anatomical Therapeutic Chemical (ATC) classification codes:<br>01454M—Goserelin<br>02722G—Medroxyprogesterone 10 mg × 100 tablets<br>02962X—Nafarelin
Hospital/Emergency Department	Reported once or more:<br>International Classification of Diseases (ICD) version and diagnosis codes:<br>ICD-9-CM 617.0–617.9 Endometriosis<br>ICD-10-AM N80.0–N80.9 Endometriosis
