# Supplementary material for: First Australian estimates of incidence and prevalence of uterine fibroids: a data linkage cohort study 2000–2022
Source: Hum Reprod. 2024 Jul 16;39(9):2134–43. doi: 10.1093/humrep/deae162 (PMC11373412; doi:10.1093/humrep/deae162)
Supplement: deae162_Supplementary_Table_S3 [file deae162_supplementary_table_s3.pdf]

OXFORD
human reproduction
Human Reproduction, 2024, 1–1
https://doi.org/10.1093/humrep/deae162
Supplementary Data

Supplementary Table S3. Estimated prevalence of uterine fibroids, by 5-year age group in the 1973–1978 cohort of the Australian Longitudinal Study on Women's Health (n = 8066).

Age (years)	Uterine fibroid cases	N	Estimated prevalence (%)	95% CI
20–24	<10	6872	0.01	0.00–0.03
25–29	13	8066	0.16	0.13–0.21
30–34	97	8066	1.12	1.02–1.24
35–39	245	8066	3.01	2.83–3.19
40–44	441	8058	5.43	5.20–5.68
45–49	324	4372*	7.27	6.90–7.64

Percentages are weighted by area of residence.
* Overall N is smaller because not every woman had reached this age group by study end.
N, number of women.
