# Supplementary material for: First Australian estimates of incidence and prevalence of uterine fibroids: a data linkage cohort study 2000–2022
Source: Hum Reprod. 2024 Jul 16;39(9):2134–43. doi: 10.1093/humrep/deae162 (PMC11373412; doi:10.1093/humrep/deae162)
Supplement: deae162_Supplementary_Table_S4 [file deae162_supplementary_table_s4.pdf]

OXFORD
human reproduction

Human Reproduction, 2024, 1–1
https://doi.org/10.1093/humrep/deae162
Supplementary Data

Supplementary Table S4. Age-specific incidence of uterine fibroids in the 1973–1978 cohort of the Australian Longitudinal Study on Women's Health (n = 8066).

Age (years)	Uterine fibroid cases	Person-years	Incidence rate per 1000 person-years	95% CI
20–24	<10	31 933.7	0.06	0.02–0.25
25–29	28	40 280.4	0.70	0.48–1.01
30–34	115	39 898.0	2.88	2.40–3.46
35–39	179	39 197.1	4.57	3.95–5.29
40–44	189	38 119.8	4.96	4.30–5.72
45–49	52	16 652.2	3.12	2.38–4.10
**Total**	**565**			
