# Supplementary material for: First Australian estimates of incidence and prevalence of uterine fibroids: a data linkage cohort study 2000–2022
Source: Hum Reprod. 2024 Jul 16;39(9):2134–43. doi: 10.1093/humrep/deae162 (PMC11373412; doi:10.1093/humrep/deae162)
Supplement: deae162_Supplementary_Table_S6 [file deae162_supplementary_table_s6.pdf]

OXFORD
human reproduction
Human Reproduction, 2024, 1–1
https://doi.org/10.1093/humrep/deae162
Supplementary Data

Supplementary Table S6. Age-specific incidence of uterine fibroids in the 1973–1978 cohort of the Australian Longitudinal Study on Women's Health (n = 8066) in a sensitivity analysis that additionally included women who only reported a diagnosis/treatment of uterine fibroids by survey.

Age (years)	Uterine fibroid cases	Person-years	Incidence rate per 1000 person-years	95% CI
20–24	<10	31 933.7	0.06	0.01–0.25
25–29	28	40 280.4	0.70	0.48–1.01
30–34	115	39 898.0	2.88	2.40–3.46
35–39	229	39 091.6	5.86	5.15–6.67
40–44	276	37 693.8	7.31	6.51–8.24
45–49	97	16 257.9	5.97	4.89–7.27
**Total**	**747**			
