# Supplementary material for: First Australian estimates of incidence and prevalence of uterine fibroids: a data linkage cohort study 2000–2022
Source: Hum Reprod. 2024 Jul 16;39(9):2134–43. doi: 10.1093/humrep/deae162 (PMC11373412; doi:10.1093/humrep/deae162)
Supplement: deae162_Supplementary_Table_S7 [file deae162_supplementary_table_s7.pdf]

OXFORD
human reproduction

Human Reproduction, 2024, 1–1
https://doi.org/10.1093/humrep/deae162
Supplementary Data

Supplementary Table S7. Rate ratios (RRs) and 95% CIs for the associations between socioeconomic factors, symptoms, reproductive factors, health factors, and health service use and incidence of uterine fibroids (2003–2022) in the 1973–1978 cohort of the Australian Longitudinal Study on Women's Health under the primary analysis and multiple imputation.

		Primary analysis RR (95% CI)[a]	Imputed analysis RR (95% CI)[a]
Age (years)		1.06 (0.99, 1.12)	1.08 (1.02, 1.14)
Area of residence	Major cities	Ref.	Ref.
	Inner regional	1.03 (0.84, 1.27)	1.07 (0.88, 1.30)
	Outer regional/rural/remote	0.95 (0.73, 1.24)	0.93 (0.72, 1.19)
Highest qualification level	Degree or higher	Ref.	Ref.
	Trade/diploma	1.05 (0.84, 1.30)	1.11 (0.91, 1.36)
	High school or less	1.13 (0.88, 1.44)	1.09 (0.86, 1.38)
Country of birth × language spoken at home	English-speaking country of birth and English spoken at home	Ref.	Ref.
	European country of birth or European language spoken at home	1.13 (0.73, 1.74)	1.20 (0.82, 1.76)
	Other country of birth or other language spoken at home	1.11 (0.65, 1.89)	1.07 (0.66, 1.73)
Headaches/Migraines	Never/Rarely	Ref.	Ref
	Sometimes	1.23 (1.01, 1.50)	1.21 (0.99, 1.47)
	Often	1.36 (1.05, 1.76)	1.39 (1.09, 1.78)
Severe tiredness	Never/Rarely	Ref.	Ref.
	Sometimes	1.31 (1.06, 1.60)	1.30 (1.06, 1.59)
	Often	1.47 (1.16, 1.87)	1.47 (1.16, 1.85)
Back pain	Never/Rarely	Ref.	Ref.
	Sometimes	1.19 (0.98, 1.45)	1.18 (0.97, 1.43)
	Often	1.33 (1.02, 1.73)	1.40 (1.09, 1.79)
Leaking urine	Never/Rarely	Ref.	Ref.
	Sometimes	0.96 (0.72, 1.27)	0.97 (0.73, 1.29)
	Often	1.77 (1.21, 2.59)	1.78 (1.24, 2.55)
Constipation	Never/Rarely	Ref.	Ref.
	Sometimes	0.93 (0.72, 1.19)	0.99 (0.78, 1.26)
	Often	1.17 (0.77, 1.79)	1.22 (0.81, 1.83)
Other Bowel problems	Never/Rarely	Ref.	Ref.
	Sometimes	1.24 (0.91, 1.70)	1.27 (0.94, 1.72)
	Often	1.97 (1.37, 2.85)	1.89 (1.32, 2.69)
Heavy periods	Never/Rarely	Ref.	Ref.
	Sometimes	2.58 (2.08, 3.21)	2.42 (1.95, 3.01)
	Often	4.07 (3.29, 5.05)	3.84 (3.12, 4.74)
Painful periods	Never/Rarely	Ref.	Ref.
	Sometimes	2.70 (2.18, 3.33)	2.50 (2.04, 3.08)
	Often	3.81 (3.02, 4.83)	3.56 (2.83, 4.48)
Smoking status	Non-smoker	Ref.	Ref.
	Former smoker	1.02 (0.83, 1.26)	1.05 (0.86, 1.28)
	Current smoker	1.13 (0.86, 1.48)	1.03 (0.79, 1.34)
BMI	$<25\ kg/m^2$	Ref.	Ref.
	$25–29.9\ kg/m^2$	1.06 (0.85, 1.34)	1.12 (0.90, 1.40)
	$\geq 30\ kg/m^2$	1.43 (1.15, 1.78)	1.46 (1.19, 1.79)
Age at menarche	≤11 years	1.47 (1.12, 1.95)	1.36 (1.05, 1.76)
	12 years	Ref.	Ref.
	13 years	0.84 (0.65, 1.08)	0.78 (0.62, 0.99)
	>13 years	0.73 (0.56, 0.96)	0.65 (0.51, 0.84)
Number of births	No births	1.36 (1.06, 1.74)	1.29 (1.04, 1.62)
	1 birth	1.35 (1.03, 1.78)	1.30 (1.01, 1.66)
	2 births	Ref.	Ref.
	3 or more births	0.91 (0.79, 1.21)	0.88 (0.68, 1.14)
Current oral contraceptive use	No	Ref.	Ref.
	Yes	0.98 (0.79, 1.21)	0.96 (0.77, 1.18)
Endometriosis	No	Ref.	Ref.
	Yes	2.03 (1.60, 2.57)	2.00 (1.61, 2.50)
Polycystic Ovarian Syndrome	No	Ref.	Ref.
	Yes	1.63 (1.22, 2.18)	1.66 (1.27, 2.17)
Low iron levels	No	Ref.	Ref.
	Yes	1.84 (1.50, 2.25)	1.85 (1.52, 2.25)

(continued)

Supplementary Table S7. Continued

		Primary analysis RR (95% CI)[a]	Imputed analysis RR (95% CI)[a]
Self-rated health	Excellent/Very good	Ref.	Ref.
	Good	1.55 (1.27, 1.88)	1.48 (1.23, 1.79)
	Fair/poor	2.25 (1.73, 2.93)	2.13 (1.65, 2.75)
Annual GP visits	Less than 2 visits	Ref.	Ref.
	2–3 visits	1.70 (1.28, 2.26)	1.60 (1.25, 2.06)
	4–6 visits	1.76 (1.31, 2.38)	1.56 (1.19, 2.04)
	More than 6 visits	1.88 (1.42, 2.48)	1.74 (1.36, 2.23)
Annual gynaecologist visits	No visits	Ref.	Ref.
	1 or more visits	1.34 (1.08, 1.68)	1.27 (1.04, 1.56)

[a] Adjusted for age, wave, and area of residence.
RR, rate ratios.

© The Author(s) 2024. Published by Oxford University Press on behalf of European Society of Human Reproduction and Embryology.
This is an Open Access article distributed under the terms of the Creative Commons Attribution License (https://creativecommons.org/licenses/by/4.0/), which permits unrestricted reuse, distribution, and reproduction in any medium, provided the original work is properly cited.
Human Reproduction, 2024, 00, 1–10
https://doi.org/10.1093/humrep/deae162
Original Article
